# The long road from person-specific models to personalized mental health treatment

**DOI:** 10.1186/s12916-020-01838-w

**Published:** 2020-12-01

**Authors:** Thomas L. Rodebaugh, Madelyn R. Frumkin, Marilyn L. Piccirillo

**Affiliations:** 1 Department of Psychological and Brain Sciences, Washington University in St. Louis, St. Louis, USA; 2 Department of Psychology, University of Washington, Seattle, USA

**Keywords:** Idiographic modeling, Personalized medicine, Mental health, Treatment, Psychotherapy

## Background

Idiographic (or person-specific) methods that examine longitudinal relationships within a single individual have become increasingly available and have generated much enthusiasm. These methods have the potential to resolve the dilemma that occurs when a treatment provider is tasked with making treatment decisions for an individual patient or client based on group-level findings (referred to in the psychological literature as the therapist’s dilemma [1, 2]). The hope is that such idiographic methods would allow the determination of the best course of treatment, by taking what is already known about the efficacy of interventions and adding knowledge about associations between a specific individual’s symptoms. For example, a clinician who is choosing between two or more treatments might find it helpful to know how symptoms are related for the individual they are preparing to treat.

## Person specific networks (PSNs) and psychological treatment

The authors of the current article [3] advance the argument that clinicians can use idiographic techniques productively and responsibly in the context of psychotherapy. Although we share this general goal, we provide further context for the challenges that currently limit its implementation.

We have advocated for the use of idiographic models [1, 2], have employed them in a research context [4], and have pilot tested their use in our clinic [5]. Our experience thus far is that both researchers and clinicians find it challenging to understand, fit, and interpret idiographic models, whether they are handled as PSNs or in one of the other frameworks available [2]. We found that, even in a clinic of student therapists who have seen repeated presentations about idiographic modeling, fewer than half thought the PSNs of their clients would inform their treatment [5]. This is in keeping with previous research evaluating the implementation of idiographic models [6] and of treatment-based algorithms more broadly [7]. Further cause for pessimism is the finding that researchers who specialize in this kind of modeling show very little agreement on how to analyze or interpret even one individual’s data [8]. This is at least in part because there are several distinct ways of analyzing idiographic data, not all of which result in PSNs per se. Of course, this does not mean that clinicians cannot use these models, or that treatment will not benefit from them. However, it does reduce our optimism regarding how widely clinicians will adopt these methods as they currently stand.

Some readers might believe that if a technique is not ready for the bedside, more work needs to be done at the bench. We think the current piece offers a useful alternative perspective, in that pure benchwork on these methods may be far less tractable than bedside and translational work. On the basic research side, an essential question is to what extent idiographic models correspond to the actual processes underlying the data. This is a difficult task. Somewhat less difficult to determine would be whether specific idiographic models are helpful in the treatment process. One example of a very basic study of this type is one in which some clinicians receive PSNs derived from their clients’ data, and others receive sham PSNs, not drawn from their clients’ data. The idea of a sham model might raise ethical concerns for some readers, but it is worth remembering that, given our lack of knowledge about what idiographic models actually capture, no idiographic model may really be superior to a sham model for treatment purposes. The question is then whether the client benefits more from treatment informed by a real model versus a sham model. Such an experiment would allow causal inference regarding whether idiographic models drawn from the client’s data are more useful than the ones not drawn from the client’s data. Of course, it would not tell us why such models are useful, or how clinicians use them; further work would be needed to answer those questions.

We believe that basic science and clinical research on idiographic models are only likely to be fruitful if continual dialog occurs bridging these two viewpoints. For example, although it is ideal to measure as many relevant constructs as possible, idiographic models can accommodate a limited number of variables. In choosing these variables, it will be important for basic scientists to understand how clinicians intend to use the models. If clinicians intend to use idiographic models to guide treatment decisions, then models must be relevant to those decisions, which typically means that variables must be included that can ultimately be intervened on using therapeutic techniques. Researchers who focus on idiographic models will need dialog with treatment providers and clinical researchers to focus their development efforts on questions that are of utility to clinicians.

We expect the most rapid progress in the use of PSNs and idiographic models more generally to occur in the contexts in which researchers are working directly with clinicians. Some examples include university psychological clinics and clinics at academic medical centers. Implementation research involving key stakeholders—clients, clinicians, and researchers—will clarify what questions need to be answered and what methods are feasible and acceptable to use. Computational models may offer a suitable way for researchers and clinicians to collaborate moving forward (see [9] for an example). We look forward to a future when these techniques, if demonstrated to be useful, are more widely available to clinicians, perhaps through automation of analysis or training artificial intelligence modules to aid in the administration of measures and interpretation of results.

## Conclusion

Personalized idiographic models provide a potential avenue toward personalized medicine, but implementation is currently limited in the majority of healthcare settings. We encourage clinicians who are interested in using these measures to seek out partnerships with researchers to maximize the impact of advances in this field.

## Data Availability

Not applicable.

## References

[1] Piccirillo ML, Beck ED, Rodebaugh TL (2019). A clinician’s primer for idiographic research: considerations and recommendations. Behav Ther.

[2] Piccirillo ML, Rodebaugh TL. Foundations of idiographic methods in psychology and applications for psychotherapy. Clin Psychol Rev. 2019;71: 90–100. 10.1016/j.cpr.2019.01.002. 10.1016/j.cpr.2019.01.002 PMC11130566 30665765

[3] von Klipstein L, Riese H, van der Veen DC, Servaas MN, Schoevers RA. Using person-specific networks in psychotherapy: challenges, limitations, and how we could use them anyway. BMC Med. 2020. 10.1186/s12916-020-01818-0. 10.1186/s12916-020-01818-0 PMC7682008 33222699

[4] Frumkin MR, Haroutounian S, Rodebaugh TL (2020). Examining emotional pain among individuals with chronic physical pain: nomothetic and idiographic approaches. J Psychosom Res.

[5] Frumkin MR, Piccirillo ML, Beck ED, Grossman JT, Rodebaugh TL. Feasibility and utility of idiographic models in the clinic: a pilot study. Psychother Res. 2020:1–15. https://www.tandfonline.com/doi/abs/10.1080/10503307.2020.1805133. 10.1080/10503307.2020.1805133 PMC7902742 32838671

[6] Zimmermann J, Woods WC, Ritter S, Happel M, Masuhr O, Jaeger U, Spitzer C, Wright AGC (2019). Integrating structure and dynamics in personality assessment: first steps toward the development and validation of a personality dynamics diary. Psychol Assess.

[7] Wisdom JP, Bielavitz S, McFarland B, Collins JC, Hamer A, Haxby D, Pollack DA (2008). Preparing to implement medication algorithms: staff perspectives and system infrastructure. J Psychiatr Pract.

[8] Bastiaansen JA, Kunkels YK, Blaauw FJ, Boker SM, Ceulemans E, Chen M, Chow SM, de Jonge P, Emerencia AC, Epskamp S (2020). Time to get personal? The impact of researchers choices on the selection of treatment targets using the experience sampling methodology. J Psychosom Res.

[9] Burger J, van der Veen DC, Robinaugh DJ, Quax R, Riese H, Schoevers RA, Epskamp S (2020). Bridging the gap between complexity science and clinical practice by formalizing idiographic theories: a computational model of functional analysis. BMC Med.

